# Factors Influencing Global Health Related Quality of Life in Elderly Cancer Patients: Results of a Secondary Data Analysis

**DOI:** 10.3390/geriatrics3010005

**Published:** 2018-01-30

**Authors:** Heike Schmidt, Thomas Nordhausen, Stephanie Boese, Dirk Vordermark, Sally Wheelwright, Andreas Wienke, Colin D. Johnson

**Affiliations:** 1 Institute of Health and Nursing Sciences, Medical Faculty, Martin Luther University Halle-Wittenberg, Magdeburger Str. 8, 06112 Halle (Saale), Germany; thomas.nordhausen@uk-halle.de (T.N.); Stephanie.Boese@bergmannstrost.de (S.B.); 2 Department of Radiation Oncology, University Hospital Halle (Saale), Medical Faculty, Martin Luther University Halle-Wittenberg, Ernst-Grube-Str. 40 06120 Halle (Saale), Germany; dirk.vordermark@uk-halle.de; 3 Health Sciences, University of Southampton, SO17 1BJ Southampton, UK; S.J.Wheelwright@soton.ac.uk; 4 Institute for Medical Epidemiology, Biostatistics, and Informatics, Medical Faculty, Martin Luther University Halle-Wittenberg, Magdeburger Str. 8, 06112 Halle (Saale), Germany; andreas.wienke@uk-halle.de; 5 Cancer Sciences, University of Southampton, SO17 1BJ Southampton, UK; C.D.Johnson@soton.ac.uk

**Keywords:** geriatric oncology, health related quality of life, social function, fatigue, burden of illness, supportive care

## Abstract

Cancer treatment for elderly patients is often complicated by poor physical condition, impaired functioning and comorbidities. Patient reported health related quality of life (HRQOL) can contribute to decisions about treatment goals and supportive therapy. Knowledge about factors influencing HRQOL is therefore needed for the development of supportive measures and care pathways. An exploratory secondary data analysis on 518 assessments of the European Organisation for Research and Treatment of Cancer (EORTC) core questionnaire (EORTC QLQ-C30) and the elderly module (EORTC QLQ-ELD14) was performed to identify factors predictive for global HRQOL. Preliminary simple and multivariable regression analyses were conducted resulting in a final model comprising sociodemographic and disease specific variables and scales of the QLQ-C30 and QLQ-ELD14. Age, sex and disease related variables explained only part of the variance of global HRQOL (adjusted R^2^ = 0.203). In the final model (adjusted R^2^ = 0.504) fatigue, social function, burden of illness and joint stiffness showed possible influence on global HRQOL. Fatigue, social function and burden of illness seem to have the largest impact on global HRQOL of elderly cancer patients. Further prospective studies should examine these domains. Actionable symptoms should be given special attention to initiate targeted supportive measures aiming to maximize HRQOL of older cancer patients.

## 1. Introduction

As new therapeutic options improve survival rates in cancer [1], the maintenance of quality of life (QOL) has become a major therapeutic goal. In this context, it is important to recognize that elderly cancer patients as a group are more heterogeneous than younger patients with respect to their physiological reserve, functional impairments and comorbidities [2,3]. Therefore, in order to detect relevant risk factors and resources and to adjust the therapy accordingly, a comprehensive geriatric assessment (CGA) comprising mobility, cognition, nutritional status and psychosocial aspects is recommended [3]. In addition to the medical condition, patients’ individual preferences should be taken into account in the process of decision-making [4]. In this context, maintenance of health related quality of life (HRQOL), which encompasses the effects of health, illness and treatment on QOL [5], independence and the ability to perform normal activities are key issues reported by cancer patients of all age groups [6]. In childhood, good self-esteem and scholarship are identified as key elements, in adulthood social support becomes fundamental and in the elderly activities assume a significant value [7,8,9]. In comparison to younger people with cancer, older people with cancer tend to prefer a better HRQOL to an increased length of life [9]. Therefore, maintenance of HRQOL and functioning are integral parts of cancer care for older patients [10] and factors like the fear of side effects, treatment discomfort and the fear of losing independence are important aspects for accepting or declining cancer therapy [11]. These issues should be considered in the decision-making process and throughout the treatment trajectory. Targeted interventions and adequate measurement tools assessing the effects of supportive therapy and these interventions on HRQOL should be developed and utilized. To achieve these goals, a sound understanding of HRQOL in elderly cancer patients, of possible influencing factors and of differences in comparison to younger patients is required. 

The HRQOL model developed by Ferrans et al. [5] that is based on Wilson and Cleary’s model [12] includes the possible influence of biological and demographic factors and characteristics of the social environment on HRQOL. The model underlines the subjective nature of health perceptions and describes the impact of symptoms on levels of activity and functioning [5]. This individual perception may vary considerably. For some patients, symptoms or functional restrictions may cause a reduction in perceived global HRQOL but not all patients with poor global HRQOL might experience these problems. There may be interdependencies like depressed mood influencing the perception of HRQOL and poor HRQOL possibly influencing the state of mind [13]. Furthermore, in the process of coping with their disease, patients might adapt their assessment of HRQOL to their changed condition, a phenomenon described as response shift [14,15,16,17], which might explain the finding, that global HRQOL scores of cancer patients are often comparable to the general population [18]. Although global HRQOL scores may be less precise than specific scales in detecting group differences over time [19] they are still informative if the aim is to optimize cancer care and to support patients’ adjustment and coping with the disease. Therefore, it is of interest to explore which factors predict global HRQOL to optimize supportive therapy for elderly cancer patients. 

Factors which could influence HRQOL may be potentially modifiable (e.g., patient-reported symptoms like pain or functional impairments), or unmodifiable like sociodemographic variables (e.g., age, education, gender) or disease related variables (e.g., treatment intention, disease stage, and comorbidities). Recent studies have revealed for instance the impact of comorbidities on HRQOL in elderly patients with multiple myeloma [20] and the negative impact of cancer related fatigue on global HRQOL in cancer patients [21]. For patients with advanced cancer, emotional functioning, pain, appetite loss [22] and social support [23] have been shown to influence HRQOL. However, there are few studies comparing age related differences of HRQOL [24,25,26] and examining HRQOL in older cancer patients specifically. Therefore, this study aimed to explore which factors are predictive of global HRQOL in older cancer patients in order to formulate hypotheses for future prospective studies and to gather information to inform the development of supportive measures targeting modifiable factors and care pathways intended to improve HRQOL. 

## 2. Material and Methods

An exploratory secondary data analysis of 518 one-time assessments of the European Organization for Research and Treatment of Cancer (EORTC) Quality of life Questionnaire (EORTC QLQ-C30) and the elderly module (EORTC QLQ-ELD14) was performed to examine which unmodifiable and modifiable factors predicted global HRQOL. Based on the models of Ferrans [5] and Wilson and Cleary [12] unmodifiable factors are sociodemographic characteristics of the individual e.g., age, sex and clinician-reported disease related factors like disease stage, treatment intention and comorbidities. Modifiable factors comprise patient-reported symptoms and modifiable functional restrictions. While there may be interactions and interdependencies between symptoms and functional restrictions, definite hierarchical causal relationships have not yet been comprehensively proven. Therefore, we used an exploratory non-hierarchical regression model including sociodemographic and disease related factors and subscales of the EORTC QLQ-C30 and QLQ-ELD14 representing mostly modifiable factors.

The primary data had been collected for the validation of the EORTC QLQ-ELD14 [27]. Patients eligible for this study were aged >70 years and had a confirmed diagnosis of any primary, recurrent or metastatic cancer. Further details are given in the results paragraph and are described in the publication by Wheelwright et al. [27]. For the original study, ethical and research governance approvals were obtained at each centre in accordance with local requirements and all patients provided written informed consent. For the secondary data analysis, the data set was provided by the data repository of the EORTC Quality of Life Group.

### 2.1. HRQOL Questionnaires 

The EORTC QLQ-C30 comprises five functioning scales (physical, role, cognitive, emotional and social), three symptom scales (fatigue, pain, nausea and vomiting), six single items assessing frequent symptoms (dyspnea, appetite loss, sleep disturbance, constipation, diarrhea and perceived financial burden) and a global health status/quality of life scale [28]. All scale and item scores are linearly transformed to a 0–100 scale, higher scores representing better functioning but worse symptom burden [29]. The EORTC QLQ-ELD14 questionnaire comprises five scales (mobility, worries about others, future worries, maintaining purpose and burden of illness) and two single items (joint stiffness and family support). As for the QLQ-C30 all scale and item scores are transformed to a 0–100 scale, higher scores representing a worse outcome except for maintaining purpose and family support [27]. 

### 2.2. Statistical Analyses

Regression analyses were carried out to investigate the associations between global HRQOL and sociodemographic factors, clinician-reported disease related factors and patient-reported factors (subscales of HRQOL). Comparable to other studies [22,30,31], subscales of the HRQOL questionnaires were included to examine the possible impact of patient-reported and potentially modifiable factors e.g., symptoms and functioning on global HRQOL in this sample of elderly cancer patients. 

The analyses comprised preliminary simple regression analyses and multivariable regression analyses. Preliminary simple analyses were performed in order to identify unmodifiable socio-demographic and disease related factors possibly influencing global HRQOL. Global HRQOL, as assessed by questions 29 and 30 of the EORTC QLQ-C30, was defined as the dependent variable. Based on theoretical considerations, 41 variables of the available socio-demographic and medical characteristics were analysed. None of these variables were considered as mediators in the sense of Ferrans et al. [5]. In order to facilitate analyses, ordinal variables were dichotomized (e.g., education low vs. high, ECOG good (0–2) vs. poor (3, 4). In addition to the preselected variables age and sex, all variables with *p* < 0.05 were included into the multivariable regression analyses. Variables representing items of the G8 screening tool, a validated screening tool considering eight questions for identifying problems of older patients [32], are labelled (G8) accordingly, as the instrument was used for data collection in the primary work [27]. 

Unmodifiable factors like sociodemographic characteristics of the individual e.g., age, sex and clinician-reported disease related factors like disease stage, treatment intention and comorbidities and patient-reported potentially modifiable factors e.g., symptoms and functioning scales were included stepwise into the model to examine possible influence on global HRQOL. 

The first step (model 1) comprised the following socio-demographic and disease related factors identified in the preliminary simple analyses: age, sex, disease stage, disease progression, treatment intention, ECOG-Status, toxicity level, food intake (G8), weight loss (G8), >three medications/day (G8) and Charlson Comorbidity Index. 

In the second step (model 2), the EORTC QLQ-C30 symptom scales were added to the model. In the third step EORTC QLQ-C30 functioning scales were added (model 3) and in the fourth and final step the EORTC QLQ-ELD14 scales were added (model 4).

After visual inspection of the distribution of global HRQOL data, no major deviations from symmetry were observed and therefore linear regression was deemed appropriate. We investigated the correlation between the variables based on their tolerance values (appendix A and B, available online). In all models the tolerance values were >0.2. Consequently, multicollinearity is not a major issue in the models [33]. As missing values were not imputed and only patients with complete data with respect to all variables were included in the multivariable regression analyses *n* = 341 complete data sets remained for the final analysis. For better comparability of the results, all models were carried out with the final sample of *n* = 341 patients. Sociodemographic, medical characteristics and HRQOL scores of both samples were descriptively contrasted to examine comparability of the reduced sample with the study population.

## 3. Results

### Study Population

The mean age of the participants of the whole sample (*n* = 518) was 77.3 years (SD: 4.9), 48.8% were male and 83.3% had a good ECOG performance status between 0 and 2. The subsample included in the multivariable regression comprised the reduced number of *n* = 341 participants. Mean age of these participants was 77.0 years (SD: 4.7), 50.7% were male and 91.5% had an ECOG status between 0 and 2. Sociodemographic data for both samples are summarized in Table 1, disease and therapy-related data in Table 2. Unadjusted mean values of all HRQOL scales for both samples are shown in Table 3.

The results of the multivariable regression models are described briefly in the following paragraphs.

The results of all models are summarized in Table 4.

Model 1 regression analysis including unmodifiable and disease related variables with respect to global HRQOL (EORTC QLQ-C30)

In this model EGOG status regression coefficient (β = −8.78, 95%CI: −16.704; −0.855), food intake (G8) (β = 13.915, 95%CI: 8.398; 19.431) and (more than) three medications (G8) (β = 8.239, 95%CI: 3.879; 12.599) showed a possible influence on global HRQOL. Adjusted R^2^ of this model is 0.203, indicating that 20,3% of the variation of global HRQOL could be explained with the variables included.

As an example for the interpretation of the regression coefficient of dichotomous variables, for ECOG status β = −8.78 means that the mean value of global HRQOL of patients with poor ECOG status (3,4) is 8.8 less compared to patients with good ECOG status. As β is a point estimate of the true effect in the population, the confidence interval shows the true value is located within this interval with 95% probability. 

Model 2 regression analysis including unmodifiable and disease related variables and EORTC QLQ-C30 symptom scales with respect to global HRQOL (EORTC QLQ-C30)

In this model (more than) three medications (G8) (β = 4.941, 95%CI: 1.286; 8.596), fatigue (β = −0.365, 95%CI: −0.463; −0.267), pain (β = −0.097, 95%CI: −0.180; −0.014) and financial problems (β = −0.116, 95%CI: −0.198; −0.034) showed a possible influence on global HRQOL. Adjusted R^2^ of this model is 0.460, indicating that 46% of the variation of global HRQOL could be explained with the variables included.

As an example for the interpretation of the regression coefficient of continuous variables, for fatigue β = −0.365 means that an increase of fatigue by one point is associated with a mean decrease of global HRQOL by 0.4 points.

Model 3 regression analysis including unmodifiable and disease related variables, EORTC QLQ-C30 symptom scales and EORTC QLQ-C30 functioning scales with respect to global HRQOL (EORTC QLQ-C30)

In this model fatigue (β = −0.243, 95%CI: −0.353; −0.134), pain (β = −0.083 95%CI: −0.164; −0.002), physical function (β = 0.173, 95%CI: 0.041; 0.305) and social function (β = 0.137, 95%CI: 0.054; 0.221), showed a possible influence on global HRQOL. Adjusted R^2^ of this model is 0.497, indicating that 49.7% of the variation of global HRQOL could be explained with the variables included.

Model 4 regression analysis including unmodifiable and disease related variables, EORTC QLQ-C30 symptom scales, EORTC QLQ-C30 functioning scales and EORTC QLQ-ELD14 scales with respect to global HRQOL (EORTC QLQ-C30)

In this final model, only fatigue (β = −0.223, 95%CI: −0.334; −0.112), social function (β = 0.099, 95%CI: 0.008; 0.191), joint stiffness (β = −0.064, 95%CI: −0.126; −0.002), and burden of illness (β = −0.071, 95%CI: −0.141; −0.001) showed a possible influence on global HRQOL. In conclusion, it can be stated that in this model fatigue prevailed, the regression coefficient β of -0.223 meaning, that an increase of fatigue by a clinically relevant amount of 10 points on a 0–100 scale [34] will be correlated with a decrease of global HRQOL of 2.2 points. Adjusted R^2^ of this model is 0.504, indicating that 50,4% of the variation of global HRQOL could be explained with the variables included.

## 4. Discussion

This study explored factors that might influence global HRQOL in elderly cancer patients in a large international data set representative of elderly cancer patients with a wide range of cancers [27]. Based on the model of Ferrans [5], unmodifiable factors and potentially modifiable factors like symptoms were included stepwise into the analyses. It is important to note that the chosen method can only examine associations and causal relationships cannot be claimed. The stepwise approach for multivariable regression analyses resulted in a final model showing that fatigue, social functioning and burden of illness had the strongest association and possible influence on global HRQOL. These findings are in line with other research describing fatigue and social functioning as important contributors to HRQOL in cancer patients [21] and support the models of Ferrans [5] and Wilson and Cleary [12] that describe symptoms and functioning as factors possibly influencing HRQOL although causality cannot be proven.

Although the final model has the best fit, explaining 50.4% of the variance of global HRQOL, the preceding models provide useful insights. In model 2, when EORTC QLQ-C30 symptom scales were added, fatigue, pain and financial problems were identified as factors possibly influencing global HRQOL. The use of more than three medications remained in the model. For older cancer patients, the use of more than three medications per day might represent health problems with a possible impact on overall wellbeing and HRQOL and a possible risk for polypharmacy. This finding underlines the importance of a medication review for older patients including all self-administered medication [35].

Adding EORTC QLQ-C30 functioning scales in model 3, fatigue, physical function, social function and pain prevailed. The importance of these symptoms and functioning domains is also supported by previous studies examining HRQOL of cancer patients [21,22,31]. In the final model, when the EORTC QLQ-ELD14 scales were added, fatigue still showed the strongest possible impact on global HRQOL followed by social function, burden of illness and joint stiffness. With respect to pain and physical function, it can be presumed that the more comprehensive questions of the EORTC QLQ-ELD14 about burden of illness and burden of treatment encompass the content of these items. The finding that joint stiffness prevailed while pain left the model might be understandable as trouble with joints e.g., stiffness or pain can be incapacitating and hamper mobility, while pain in general might be represented by burden of illness. This finding is supported by a study on HRQOL in elderly multiple myeloma patients describing bone aches, and pain in hips, arms and shoulders having a negative impact on global health status [30]. 

As the interference of symptoms with functioning is especially burdensome for elderly patients and can lead to distress [25], regular assessment of symptoms and functioning is advisable to trigger timely targeted supportive care. In addition, attention should be given to other factors like social support, particularly in the case of limited functional capacity. In patients with advanced cancer, Rodriguez et al. found social support to be the most important contributor of overall HRQOL [23]. The availability of social support can play a major role in the upkeep of medical appointments and social relations for patients with disease related impairments. Therefore, an assessment of psychosocial risk factors and the development of targeted interventions e.g., psychological interventions on families could prove beneficial in the endeavor to optimize supportive care. 

In conclusion, it can be stated that the association between fatigue, social functioning and burden of illness with global HRQOL in our data underlines the significance of patient-reported outcomes for the treatment of older cancer patients. Particular attention should be given to symptoms that affect HRQOL which are sensitive to treatment or supportive measures to facilitate targeted supportive care. Special attention should also be given to individual and environmental characteristics that might influence social function and the use of health services. 

### 4.1. Limitations of the Study

This study has several limitations. The adjusted R^2^ value of around 0.5 indicates that there are additional factors (not measured or possibly not yet known), which may influence the global HRQOL. The health status of the participants was generally high, as indicated by the limited number of participants with poor ECOG status, meaning generalizability might be limited. However, with the exception of emotional function and pain, which were better for the study population, the unadjusted mean scores of the EORTC QLQ-C30 did not differ significantly (defined as >10 point difference) in comparison to reported scores of cancer patients of a comparable age cohort [24]. With respect to comorbidities, the data on the use of more than three medications indicate the presence of more health problems, than captured by the Charlson comorbidity index, as might be expected in older cancer patients. These health problems might have been captured more comprehensively by the cumulative illness rating scale (CIRS). In addition, the documentation of the exact amount of prescribed and self-administered medication would have been of interest. With respect to dementia and depression, the available information as given by the respective single item of the G8 screening tool is not suitable for analyzing the influence of either impairment on HRQOL. In addition, this study has some limitations due to the data of the original study. For the original study [27] data of people who declined to take part in the study were not recorded, due to ethical considerations. A considerable number of patients (*n* = 177) had to be excluded from the analysis due to incomplete or missing data of the original study. Except for the ECOG status which was better for the subsample, the descriptive comparison of the percentages did not reveal any major differences between both groups.

### 4.2. Implications for Future Research

The variables associated with HRQOL in elderly cancer patients should be investigated in further prospective studies, which could also examine the development of HRQOL over time including response shift in connection with different diagnoses and disease trajectories. In addition to symptoms and functioning, social support [36], self-efficacy [37] and ways of coping could be investigated as possible influencing factors on adjustment to the illness and the individual rating of HRQOL. Findings should feed into the design of supportive measures and multicomponent interventions including psychosocial interventions for spouses and families aimed at maximizing the HRQOL of elderly cancer patients. 

## Figures and Tables

**Table 1:** Sociodemographic data (*n* = 518; *n* = 341 complete data sets included in regression analyses) All values are number (%) unless stated otherwise.

Sociodemographic Data	*n* = 518	*n* = 341
**Sex**		
Male	253 (48.8)	173 (50.7)
Female	264 (51.0)	168 (49.3)
Missing	1 (0.2)	0 (0)
**Age**	Mean: 77.3; SD: 4.9	Mean: 77.0; SD: 4.7
<80	365 (70.5)	246 (72.1)
80–85	118 (22.8)	78 (22.9)
>85	35 (6.8)	17 (5.0)
**Education**		
No education/primary education	187 (36.1)	136 (39.9)
Secondary education	184 (35.5)	119 (34.9)
College	88 (17.0)	50 (14.9)
University	50 (9.7)	34 (10.0)
Missing	9 (1.7)	2 (0.6)
**Employment Level**		
Unskilled	130 (25.1)	102 (29.9)
Skilled	155 (29.9)	97 (28.4)
Admin	125 (24.1)	85 (24.9)
Professional	85 (16.4)	56 (16.4)
Missing	23 (4.4)	1 (0.3)
**Living**		
Alone	131 (25.3)	78 (22.9)
With Family	359 (69.3)	278 (75.7)
Supported	8 (1.5)	5 (1.5)
Missing	20 (3.9)	0 (0)
**Children**		
No children	41 (7.9)	20 (5.9)
One or more children	459 (88.6)	321 (94.1)
Missing	18 (3.5)	0 (0)
**Carer Support**		
At home	273 (52.7)	192 (56.3)
Easily available	136 (26.3)	95 (27.9)
Not available	55 (10.6)	32 (9.4)
Carer for other	18 (3.5)	12 (3.5)
Missing	36 (7.0)	10 (2.9)

**Table 2:** Disease- and therapy-related data (*n* = 518; *n* = 341 complete data sets included in regression analyses) All values are number (%) unless stated otherwise.

Disease and Therapy Related Data	*n* = 518	*n* = 341
**Primary Cancer Localisation**		
Breast	91 (17.6)	58 (17.0)
Colorectal	87 (16.8)	63 (18.5)
Lung	63 (12.2)	52 (15.2)
Ovary	23 (4.4)	17 (5.0)
Prostate	75 (14.5)	53 (15.5)
Upper GI	21 (4.1)	14 (4.1)
Haematological	54 (10.4)	10 (2.9)
Other	104 (20.1)	74 (21.7)
**Disease stage**		
Not metastatic	306 (59.1)	212 (62.2)
Metastatic	176 (34.0)	129 (37.8)
Missing	36 (7.0)	0 (0)
**Disease Progression**		
Yes	54 (10.4)	39 (11.4)
No	464 (89.6)	302 (88.6)
**Therapy**		
Surgery	253 (48.8)	190 (55.7)
Chemotherapy	300 (57.9)	211 (61.9)
Radiotherapy	205 (39.6)	133 (39.0)
Hormonal Therapy	93 (18.0)	61 (17.9)
**Treatment Intention**		
Curative	288 (55.6)	208 (61.0)
Palliative	189 (36.5)	133 (39.0)
Missing	41 (7.9)	0 (0)
**Toxicity Level of Therapy**		
Low	484 (93.4)	313 (91.8)
Severe	34 (6.6)	28 (8.2)
Missing	36 (7.0)	0 (0)
**ECOG-Status**		
Good (0–2)	434 (83.8)	312 (91.5)
Poor (3,4)	45 (8.7)	29 (8.5)
Missing	39 (7.5)	0 (0)
**Charlson Comorbidity Index**	Mean: 0.7, SD: 1.1	Mean: 0.6, SD: 1.0
Number of comorbidities/patient	Min.: 0, Max.: 4	Min.: 0, Max.: 4
No Comorbidity (score 0)	321 (62.0)	213 (62.5)
At least one Comorbidity (score > 0)	197 (38.0)	128 (37.5)
**Frequent Comorbidities**		
Cardiovascular disease	104 (20.1)	64 (18.8)
Diabetes	72 (13.9)	46 (13.5)
Pulmonary disease	31 (6.0)	18 (5.3)
Renal failure	18 (3.5)	9 (2.6)
Liver disease	16 (3.1)	11 (3.2)
**G8 Items (dichotomized)**		
Food intake poor	172 (33.2)	118 (34.6)
Weight loss > 3 kg	122 (23.6)	88 (25.9)
Unable to leave the house	87 (16.8)	55 (16.1)
Dementia or depression	52 (10.0)	33 (9.7)
Malnutrition	54 (10.4)	40 (11.7)
>Three medications	272 (52.5)	190 (55.7)
Own health status perceived poor	180 (34.7)	128 (37.5)
Age > 80	161 (31.1)	99 (29.0)
**G8 total Score**	M: 12.5, SD: 2.9	M: 12.7, SD: 3.0
Missing	19 (3.7)	0 (0)

**Table 3:** EORTC QLQC30 and QLQ-ELD14 scales (*n* = 518; *n* = 341 complete data sets included in regression analyses).

Variable	Total Sample (*n* = 518)		Complete Data Sets Included in Analyses (*n* = 341)	
	*n*	Mean (SD)	*n*	Mean (SD)
QLQ-C30 global health status	513	65.2 (21.9)	341	65.6 (21.6)
QLQ-C30 symptom scales				
Fatigue	505	35.6 (27.5)	341	35.1 (27.3)
Nausea/vomiting	512	7.0 (15.8)	341	7.0 (15.7)
Pain	507	21.2 (27.6)	341	20.0 (25.9)
Dyspnoea	513	23.2 (30.6)	341	22.1 (30.2)
Insomnia	510	27.2 (33.1)	341	26.4 (32.7)
Appetite loss	513	18.7 (30.9)	341	18.9 (30.7)
Constipation	514	21.0 (29.0)	341	22.0 (29.1)
Diarrhoea	513	8.9 (20.5)	341	9.5 (21.5)
Financial problems	513	8.7 (21.1)	341	9.9 (22.7)
QLQ-C30 functioning scales				
Physical Function	508	73.3 (23.7)	341	74.3 (32.9)
Role Function	514	70.8 (32.8)	341	71.6 (32.4)
Emotional Function	506	82.2 (20.3)	341	82.7 (19.6)
Cognitive Function	509	83.3 (20.6)	341	84.2 (20.3)
Social Function	506	77.8 (29.4)	341	77.6 (29.1)
QLQ-ELD14 scales				
Mobility	503	28.4 (28.5)	341	27.9 (29.0)
Joint Stiffness	518	30.1 (32.4)	341	28.6 (31.8)
Family Support	484	70.7 (34.7)	341	71.0 (35.2)
Worries about others	493	39.6 (33.0)	341	41.2 (33.6)
Future worries	505	34.0 (31.9)	341	31.6 (32.5)
Maintaining purpose	511	64.6 (29.8)	341	63.7 (30.9)
Burden of illness	506	41.7 (32.7)	341	42.2 (32.6)

**Table 4:** Four stepwise regression models with respect to global HRQOL (EORTC QLQ-C30) including fixed and disease related variables, EORTC QLQ-C30 symptom scales, EORTC QLQ-C30 functioning scales and EORTC QLQ-ELD14 scales (for better comparability all models were carried out with the final sample of *n* = 341 patients).

Variable	Model 1		Model 2		Model 3		Model 4 (Final Model)	
	Regression Coefficient	Confidence Interval (95%)	Regression Coefficient	Confidence Interval (95%)	Regression Coefficient	Confidence Interval (95%)	Regression Coefficient	Confidence Interval (95%)
Fixed and disease related variables								
Age	0.170	−0.279; 0.619	−0.102	−0.478; 0.275	−0.099	−0.480; 0.281	−0.100	−0.481; 0.281
Sex	−2.992	−7.151; 1.167	1.153	−2.425; 4.732	1.615	−1.988; 5.218	1.980	−1.663; 5.624
Disease stage *	−1.951	−7.385; 3.482	1.953	−2.635; 6.540	2.134	−2.358; 6.627	2.309	−2.188; 6.805
Disease progression *	−3.93	−11.099; 3.239	−2.291	−8.258; 3.677	−2.996	−8.795; 2.802	−3.105	−8.888; 2.677
Treatment intention *	−2.071	−7.381; 3.238	−2.569	−6.972; 1.834	−1.466	−5.765; 2.832	−2.097	−6.411; 2.217
ECOG status *	−8.78	−16.704; −0.855	−0.936	−7.718; 5.845	4.087	−3.260; 11.435	4.311	−3.086; 11.708
Toxicity level *	−5.749	−13.739; 2.242	3.215	−3.624; 10.054	3.592	−3.022; 10.205	4.165	−2.464; 10.794
Food intake (G8) *	13.915	8.398; 19.431	3.997	−1.285; 9.280	2.323	−2.843; 7.490	2.507	−2.656; 7.669
Weight loss (G8) *	−1.678	−7.589; 4.234	−1.166	−6.088; 3.755	−1.032	−5.809; 3.744	−1.053	−5.890; 3.785
>Three medications (G8) *	8.239	3.879; 12.599	4.941	1.286; 8.596	3.435	−0.236; 7.107	2.922	−0.777; 6.622
Charlson Comorbidity Index *	−2.302	−6.705; 2.1	−0.268	−3.943; 3.407	−0.228	−3.838; 3.382	0.261	−3.389; 3.911
EORTC QLQ-C30 symptom scales								
Fatigue			−0.365	−0.463; −0.267	−0.243	−0.353; −0.134	−0.223	−0.334; −0.112
Nausea/vomiting			0.027	−0.100; 0.154	0.022	−0.102; 0.146	0.035	−0.090; 0.159
Pain			−0.097	−0.180; −0.014	−0.083	−0.164; −0.002	−0.067	−0.150; 0.015
Dyspnoea			0.047	−0.021; 0.116	0.051	−0.017; 0.119	0.048	−0.020; 0.117
Insomnia			−0.021	−0.078; 0.037	−0.002	−0.059; 0.054	0.002	−0.055; 0.059
Appetite loss			−0.061	−0.143; 0.020	−0.038	−0.119; 0.043	−0.049	−0.130; 0.033
Constipation			−0.034	−0.096; 0.029	−0.024	−0.085; 0.037	−0.031	−0.092; 0.029
Diarrhoea			−0.066	−0.149; 0.017	−0.060	−0.141; 0.021	−0.059	−0.140; 0.021
Financial problems			−0.116	−0.198; −0.034	−0.011	−0.102; 0.080	−0.014	−0.107; 0.079
EORTC QLQ-C30 functioning scales								
Physical Function					0.173	0.041; 0.305	0.114	−0.034; 0.262
Role Function					0.025	−0.055; 0.105	0.022	−0.059; 0.102
Emotional Function					0.040	−0.059; 0.140	0.015	−0.086; 0.116
Cognitive Function					0.020	−0.080; 0.120	−0.002	−0.103; 0.099
Social Function					0.137	0.054; 0.221	0.099	0.008; 0.191
EORTC QLQ-ELD14 scales								
Mobility							−0.035	−0.140; 0.071
Joint Stiffness							−0.064	−0.126; −0.002
Family Support							0.005	−0.050; 0.060
Worries about others							−0.006	−0.064; 0.052
Future worries							−0.005	−0.074; 0.063
Maintaining purpose							0.019	−0.041; 0.079
Burden of illness							−0.071	−0.141; −0.001
Model R^2^ (adjusted)	0.203		0.460		0.497		0.504	

* Explanation of dichotomous variables: Disease stage (not metastatic vs. metastatic), disease progression (no vs. yes), treatment intention (curative vs. palliative), ECOG status (good 0–2 vs. poor 3–4), toxicity level (low vs. severe), food intake (G8) (appetite vs. no appetite loss), weight loss (G8) (weight loss > 3 kg vs. no weight loss/weight loss < 3 kg), >three medications (yes vs. no), Charlson Comorbidity Index (no comorbidity vs. at least one comorbidity). All other variables are metric.
